# Time, action and psychosis: Using subjective time to investigate the effects of ketamine on sense of agency

**DOI:** 10.1016/j.neuropsychologia.2012.07.005

**Published:** 2013-01

**Authors:** J.W. Moore, V.C. Cambridge, H. Morgan, F. Giorlando, R. Adapa, P.C. Fletcher

**Affiliations:** a Department of Psychiatry, Brain Mapping Unit, University of Cambridge, Cambridge, UK; b Department of Psychology, Goldsmiths, University of London, London, UK; c Department of Psychiatry, University of Melbourne, Melbourne, VIC, Australia; d University Division of Anaesthesia, Addenbrooke's Hospital, Cambridge, UK

**Keywords:** Sense of agency, Time, Ketamine, Consciousness, Schizophrenia, Prodrome, Prodromal, Volition, Action

## Abstract

Sense of agency refers to the experience of initiating and controlling actions in order to influence events in the outside world. A disturbed sense of agency is found in certain psychiatric and neurological disorders, most notably schizophrenia. Sense of agency is associated with a *subjective compression of time*: actions and their outcomes are perceived as bound together in time. This is known as ‘intentional binding’ and, in healthy adults, depends partly on advance prediction of action outcomes. Notably, this predictive contribution is disrupted in patients with schizophrenia. In the present study we aimed to characterise the psychotomimetic effect of ketamine, a drug model for psychosis, on the predictive contribution to intentional binding. It was shown that ketamine produced a disruption that closely resembled previous data from patients in the early, prodromal, stage of schizophrenic illness. These results are discussed in terms of established models of delusion formation in schizophrenia. The link between time and agency, more generally, is also considered.

## Highlights

► Voluntary actions and outcomes are bound together in subjective time. ► This binding depends partly on advance prediction, something that is disrupted in schizophrenia. ► Administering ketamine, a drug model of psychosis, to healthy adults disrupted prediction. ► This effect closely resembled previous data from prodromal patients. ► Results are discussed in terms of (a) models of delusion formation and (b) the relationship between time and agency.

## 1 Introduction

In humans, voluntary goal-directed action is accompanied by an experience of initiating and controlling the action, and through it, controlling the external world. This experience is referred to as the sense of agency. A disturbance in sense of agency may lie at the heart of psychotic symptoms such as delusions and hallucinations, which are characteristic of schizophrenia - a syndrome that also entails marked alterations in the perception of time.

**Fig. 1:**
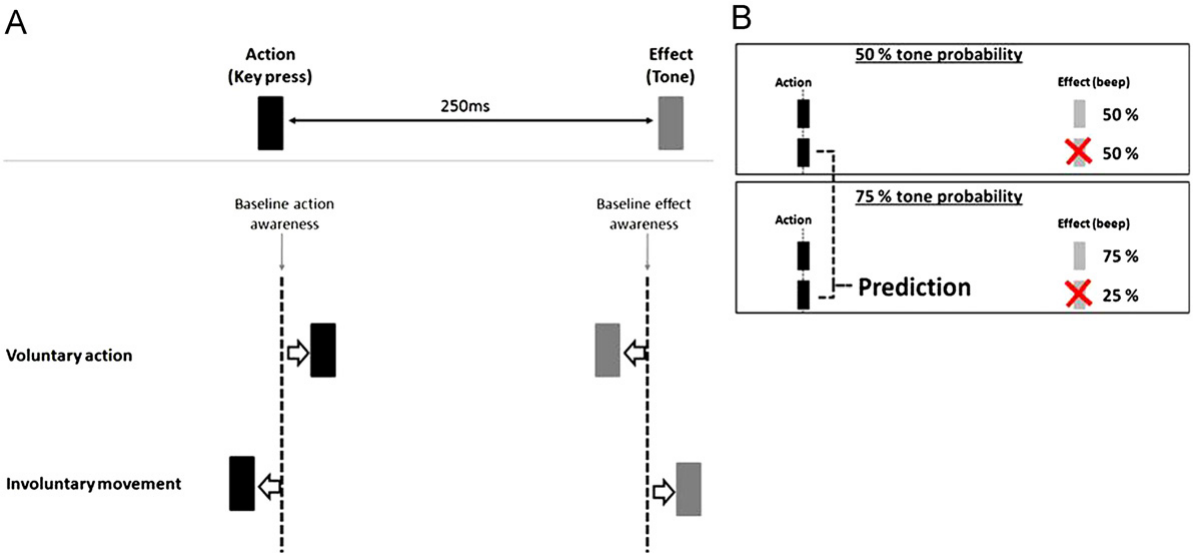
(A) The intentional binding effect. Voluntary actions and outcomes are temporally bound together in experience, whereas involuntary movements and outcomes are separated in experience (see Haggard et al., 2002). (B) Operational definition of prediction in our study. A predictive contribution to action binding was derived from subtracting the shifts in the temporal experience of action on ‘action only’ trials in 50% effect probability condition, from shifts on action ‘only trials’ in the 75% effect probability condition.

Intriguingly, this sense of agency is associated with a *subjective compression of time*, such that actions and their effects are perceived as bound together across time (Haggard, Clark, & Kalogeras, 2002; Moore & Obhi, 2012). This effect is known as ‘intentional binding’ (Fig. 1A). In the standard version of the intentional binding paradigm, participants judge the onset of either voluntary actions (a key press) or the onset of a sensory event (a tone) presented 250 ms after the action. The perceived onset of the action is shifted later in time in comparison to the perceived onset of actions in a baseline condition in which the action does not produce a tone. Furthermore, the perceived onset of the tone is shifted earlier in time relative to the perceived onset of tones in a baseline condition in which the tone is presented without action. In short, a causal action is experienced as occurring closer to the ensuing outcome while the experience of the outcome moves closer to its causal action. This binding effect is specific to *voluntary* action. When actions are *not* under voluntary control the reverse pattern of results is observed. It has therefore been proposed that intentional binding is a viable implicit measure of sense of agency (Haggard et al., 2002; Moore & Haggard, 2010; Moore & Obhi, 2012).

As noted, a disrupted sense of agency is characteristic of certain psychiatric disorders, most notably schizophrenia (Frith, 1992). Such would be the case, for example, in delusions of control, where the sufferer has a compelling sense of actions being controlled by an outside force. According to one influential model of sense of agency, the so-called ‘Comparator Model’ (CM), disordered experiences of agency in schizophrenia are produced by deficits in sensorimotor prediction. According to this view, the normal experience of agency is dependent on predictive motor control processes (Blakemore, Wolpert, & Frith, 2002; Frith, 2005). Specifically, an efference copy of motor commands is used to predict the likely sensory consequences of a voluntary action, and the comparison between these predictions and the actual sensory consequences informs sense of agency. A match between predicted and actual sensory consequences of movement promotes the feeling of *self*-agency, whereas a mismatch reduces it. According to the CM, experiences of passivity in patients with schizophrenia can be explained by impaired sensorimotor prediction during voluntary action. This impairment would lead to a faulty mismatch between the actual and expected sensory consequences. As a result, patients experience a reduced feeling of self-agency for their movements.

In support of the CM, a number of studies on sense of agency in schizophrenia have shown that patients have deficits in sensorimotor prediction (Blakemore, Smith, Steel, Johnstone, & Frith, 2000; Shergill, Samson, Bays, Frith, & Wolpert, 2005; Synofzik, Thier, Leube, Schlotterbeck, & Lindner, 2009). Compelling evidence also comes from studies using the intentional binding paradigm. Moore and Haggard (2008) confirmed the contribution of prediction to sense of agency in healthy volunteers. When actions frequently produced an outcome, the shift in perceived time of action towards the (expected tone) occurred even on rare ‘action only’ trials, on which the outcome was omitted. This suggests that *predicting* the outcome was sufficient to generate the shift in perceived time of action. This was confirmed by the reduction in binding on ‘action only’ trials in a condition where the tone was unpredictable. This approach to exploring the predictive component of intentional binding is shown in Fig. 1B: The predictive contribution represents the *difference* in binding on ‘action only’ trials in the 75% condition (where 75% of trials are followed by tones) vs. the 50% condition (where 50% of the trials are followed by tones), and the more positive this difference the greater the predictive contribution.

**Fig. 2:**
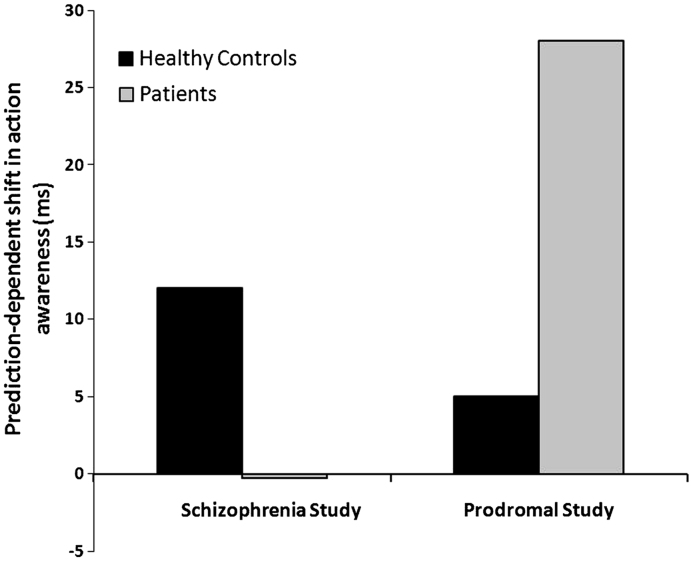
Data from previous studies on patients with schizophrenia and patients in the psychotic prodrome. These data represent the predictive contribution to action binding (i.e., the difference in binding on ‘action only’ trials in the 75% vs. 50% condition. The greater this difference the stronger the predictive contribution). Both studies replicated the predictive contribution to binding in healthy volunteers found by Moore and Haggard (2008). However, the two groups of patients showed different deficits on this task. Patients with schizophrenia showed no significant predictive contribution (from Voss et al. (2010)), whereas prodromal patients showed an excessive predictive contribution (from Hauser et al. (2011)).

Using this same procedure, deficits in sensorimotor prediction have been observed in patients with schizophrenia and in prodromal patients. However, the pattern of predictive deficits in these two groups is quite different (see Fig. 2). Patients with schizophrenia show an *absence* of predictive action binding (Voss et al., 2010), in direct support of the CM. On the other hand, prodromal patients, those who experience symptoms pointing towards a psychotic disorder but who do not yet meet diagnostic criteria, show much stronger predictive action binding relative to controls (Hauser et al., 2011).

In summary, the subjective perception of the timing of both a causal action and its ensuing outcome offers an implicit measure of SoA. Moreover, it is possible to develop this measure in order to determine the extent to which that sense emerges from a predictive relationship between an action and its consequences. This has been further refined to offer a novel way to explore the relationship between prediction, agency and timing in schizophrenia and the emerging results suggest that while, overall, disturbances in schizophrenia are compatible with disrupted SoA (as measured by altered experience of the temporal relationship between actions and outcomes), the precise nature of the disruption depends on the stage of illness and this interacts with the degree to which the action is more or less predictive of the outcome. In the current study, we sought to explore this further using a psychopharmacological study of the effects of ketamine – a drug model of early schizophrenia – on intentional binding.

Ketamine is a non-competitive NMDA receptor antagonist, which, at sub-anaesthetic levels, produces a state in healthy adults that resembles the perceptual disturbances of schizophrenia in several key ways. For example, it induces perceptual changes, ideas of reference, thought disorder and some negative symptoms (Ghoneim, Hinrichs, Mewaldt, & Petersen, 1985; Lahti, Weiler, Tamara Michaelidis, Parwani, & Tamminga, 2001; Mason, Morgan, Stefanovic, & Curran, 2008; Morgan, Mofeez, Brandner, Bromley, & Curran, 2004; Pomarol-Clotet et al., 2006). Importantly, ketamine also reproduces aberrant experiences of agency associated with schizophrenia. In a previous study using the IB paradigm it was found that the magnitude of binding in patients with schizophrenia was significantly stronger than controls (Haggard, Martin, Taylor-Clarke, Jeannerod, & Franck, 2003; Voss et al., 2010), an effect reproduced by administration of ketamine in healthy controls, where the magnitude of binding on ketamine was significantly stronger than binding in the same participants on placebo (Moore et al., 2011). Given the known neurobiological effects of ketamine, the drug model also provides a window onto the neurobiological basis of these aberrant experiences of agency.

A key issue concerning the ketamine model of psychosis concerns the stage of the disease the drug most closely resembles. Looking at the overall binding effect is unlikely to resolve this issue as augmented overall binding is associated with both established schizophrenic illness (Haggard et al., 2003; Voss et al., 2010) and the psychotic prodrome (Hauser et al., 2011). However, the aforementioned pattern of contrasting predictive impairments at different stages of the disease provides an ideal opportunity for testing this in the context of aberrant experiences of agency.

We replicated the design of the previous patient studies (Voss et al., 2010; Hauser et al., 2011) to determine the effect of ketamine on predictive action binding. If the effects of acute ketamine administration are most redolent of the established schizophrenic illness, then we would expect ketamine to reduce the predictive contribution to action binding relative to placebo. Conversely, if the effects are most redolent of the prodromal stage of the disease then we would expect there to be a significant increase in predictive action binding on ketamine relative to placebo.

We also explored the link between these putative cognitive effects of the ketamine challenge and the changes in subjective experience also arising from it. In particular we were interested in the relation between binding and changes in the experience of body perception, as measured by the clinician-administered dissociative states scale (CADSS; Bremner et al., 1998). In a previous study we found that the magnitude of the binding effect on ketamine was positively correlated with the degree of changes in body perception produced by the drug (Moore et al., 2011). In the present study we sought to replicate this effect.

## 2 Methods

### 2.1 Participants

14 participants were initially recruited to the study. Of these, 12 participants completed both ketamine and placebo sessions (8 females; mean age 23 years). The study was approved by Addenbrookes NHS Trust Research Ethics Committee. Participants provided written, informed consent.

### 2.2 Experimental design

The study used a double-blind, placebo-controlled, randomised, within-subjects design.

### 2.3 Infusion protocol

Participants were administered placebo (saline) or racemic ketamine (2 mg/mL) as an intravenous infusion using a target-controlled infusion system comprising a computer which implemented Stanpump software (S Shafer; http://www.opentci.org/doku.php?id=code:code) to control a syringe driver infusion pump (Graseby 3500; Graseby Medical Ltd, Watford, United Kingdom). Stanpump was programmed to use a 2-compartmental pharmacokinetic model (Rigby-Jones et al.), to implement a complex infusion profile designed to achieve pre-specified plasma ketamine concentrations.

During the *drug* session, participants received first low-dose ketamine (plasma target 100 ng/mL) and then higher dose (plasma target 200 ng/mL). The intentional binding task was completed at the low dose (other cognitive tasks were completed at the higher dose). We decided to run the task at the lower dose as we were mindful of the generic impairments in cognition and attention that *may* be produced by ketamine, and which may therefore have an impact on task performance.

Drug and placebo sessions were separated by at least one week. Participants also underwent a clinical assessment (see below). The order of drug and placebo visits was counterbalanced across all 12 participants (i.e., 6 participants completed the ketamine session first).

### 2.4 Intentional binding task

**Fig. 3:**
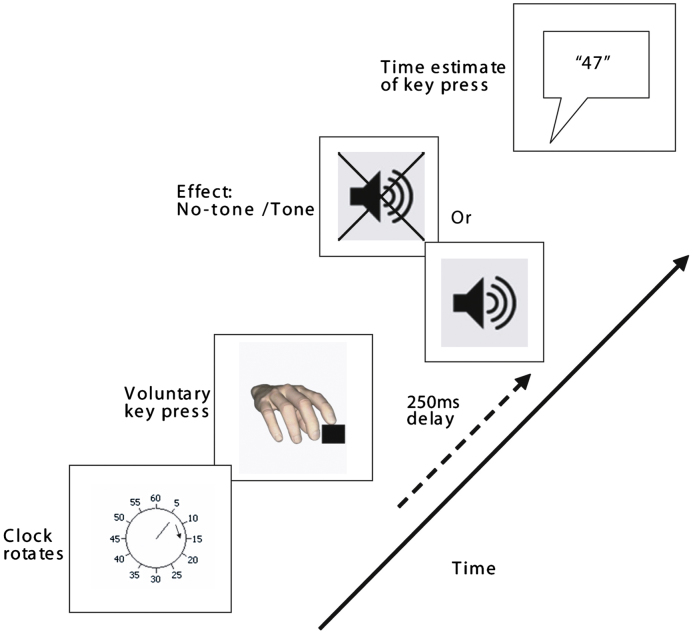
Trial structure in the *agency* condition (following Moore and Haggard (2008), Voss et al. (2010); Hauser et al., 2011). Participants pressed the key at a time of their choosing. In one condition the key pressed cause the tone on 50% of trials. In another condition, the key press caused the tone on 75% of trials. If the tone was played it was done after a delay of 250 ms from key press. Participants judged where the clock hand was when they pressed the key.

The basic trial structure is shown in Fig. 3. Participants watched a computer screen on which a hand rotated around a clock-face (marked at conventional “5-minute” intervals). Each full rotation lasted 2560 ms. There were two agency conditions: *50% outcome probability* and *75% outcome probability*. In these conditions, participants pressed a key with their right index finger at a time of their choosing. In the *50% outcome probability condition* this key press caused a tone on 50% of the trials. In the *75% outcome probability condition* this key press caused a tone on 75% of the trials. When the tone was played it was done so after a delay for 250 ms. The clock-hand then continued rotating for a random period of time (between 1500 ms and 2500 ms). When it stopped participants verbally reported the time of their key press. These judgements were blocked, so participants only made a single type of estimate on each trial in each block. To make the time estimates, participants reported the position of the hand on the clock face when they pressed the key. These two agency conditions consisted of 32 trials each.

They completed a further 32-trial *baseline* block of time estimates (*baseline action*). In this block, participants pressed the key at a time of their choosing. However, the key press never produced a tone, and on each trial participants reported the time of the key press. These baseline blocks control for individual differences in the time perception of actions. They also allow us to determine, and control for, systematic differences in the temporal experience of these events resulting from the drug. The order of these three blocks (2×agency, and 1×baseline) was randomised for each participant.

### 2.5 Data analysis: Overall action binding and predictive action binding

To calculate overall action binding, average judgment error (difference between the estimated and actual onset of action) in the baseline condition was subtracted from average judgement error across *both* outcome probability conditions, irrespective of trial type (50% and 75% conditions, ‘action only’ and ‘action+tone’ trials). The more positive the difference between these two average judgement errors the more the perceived time of action was bound towards the (putative) outcome. Following Moore et al. (2011) we predicted more overall action binding on ketamine relative to placebo.

The predictive contribution to action binding was calculated in the same way as previously described by Voss et al. (2010) and Hauser et al. (2011) (see Fig. 1B for schematic). We first calculated action binding in each outcome probability condition for both ‘action only’ and ‘action+tone’ trials. For this we subtracted the mean action judgement error in the *baseline action* condition from the mean action judgement error for each trial type in each condition. A positive value represents binding of the action towards the (putative) tone. To calculate the contribution of outcome prediction to action binding we simply subtracted the action binding score on ‘action only’ trials in the 50% outcome probability condition from the action binding score on those same trials in the 75% outcome probability condition. The resulting difference represents the contribution of outcome prediction to action binding: The more positive the difference the stronger that contribution.

Moore and Haggard (2008) have also demonstrated a postdictive contribution to action binding. This is shown by an increase in the magnitude of action binding on ‘action+tone’ trials vs. ‘action only’ trials in the 50% condition (where outcome prediction is minimal). Although we also present these data from ketamine and placebo sessions for illustrative purposes, our analyses focus only on differences in prediction. This is because deficits in prediction have been widely implicated in schizophrenia (postdiction less so) and because only differences in the magnitude of predictive influences on action binding clearly distinguish between the different stages of schizophrenic illness.

### 2.6 Clinical assessment

The Clinician-Administered Dissociative States Scale (CADSS; Bremner et al., 1998) was administered at both 100 ng/mL and 200 ng/mL. Intentional binding was run on the lower dose of 100 ng/mL (other cognitive tasks were run at the higher dose). This consists of 5 subscales: body perception, environmental perception, feelings of unreality, memory impairment, and time perception. Each subscale consists of items (questions), and participants’ responses are coded on a 5-point scale (0: “Not at all” through to 4: “Extremely”).

We focussed our analyses the ‘Body Perception’ subscale for the CADSS administered at 100 ng/mL (the infusion level at which the binding task was completed). This includes the questions: “Do you feel disconnected from your own body?” and “Does your sense of your own body feel changed: for instance, does your own body feel unusually large or unusually small?” We predicted a positive correlation between overall action binding and scores on the body perception scale (following, Moore et al., 2011).We also conducted further exploratory correlation analyses between other variables.

## 3 Results

Table 1 shows mean action binding effects and also postdictive and predictive action binding for each drug session.

### 3.1 Overall action binding and predictive action binding

We found that ketamine significantly increased the overall level of action binding (i.e., action binding averaged across trials and conditions), *t*(11)=1.83, *p*=.048 (1-tailed). This replicates a previous finding (Moore et al., 2011). This increased action binding has also been observed in patients with schizophrenia (Voss et al., 2010).

**Fig. 4:**
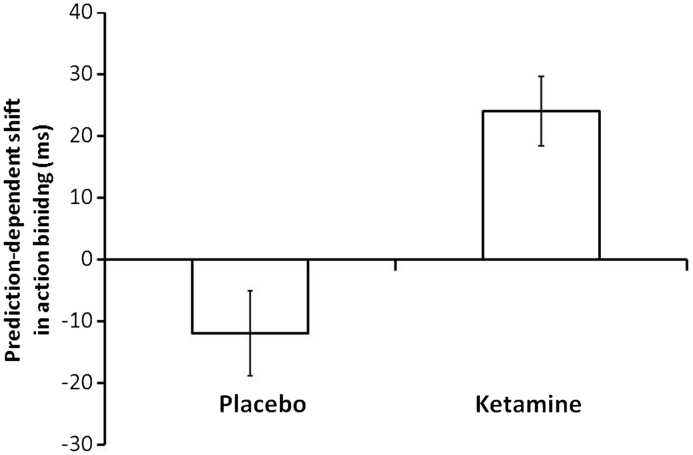
Prediction-dependent shifts in action binding (ms) on placebo and ketamine. These shifts are calculated by subtracting binding on ‘action only’ trials in the 50% condition from binding on the same trials in the 75% condition. The more positive this difference the stronger the predictive effect. Error bars represent standard error of the mean.

Of principle interest was the effect of ketamine on predictive action binding. A paired-samples *t*-test on predictive binding scores showed a significant difference between placebo and ketamine, *t*(11)=2.37, *p*=.04 (2-tailed). Inspection of Fig. 4 and Table 1 shows that ketamine engendered a significant *increase* in the predictive contribution to binding. This shows that ketamine *selectively* increased the magnitude of binding on ‘action only’ trials in the 75% condition vs. the 50% condition. This pattern of results resembles previous data in prodromal patients from Hauser et al. (2011). Furthermore, the magnitude of this predictive contribution to binding on ketamine (24 ms) was similar to that observed previously in prodromal patients (27 ms; Hauser et al., 2011).

### 3.2 Correlations between binding and CADSS scores

We also assessed the strength of correlation between these binding measures on ketamine and scores on the CADSS. A priori we expected a significant positive correlation between overall action binding on ketamine and scores on the ‘Body Perception’ sub-category of the CADSS (following Moore et al. (2011)). We also present any significant correlations following further exploratory analysis of different variables.

Overall action binding showed near-significant correlations with body perception scores (*ρ*=.47, *p*=.06; 1-tailed). Although not quite significant, the positive correlation between overall action binding and body perception scores is consistent with our results from a previous study (see Moore et al., 2011). Predictive action binding was not significantly correlated with body perception scores (*ρ*=−.41, *p*=.19; 2-tailed).

Here, we briefly present the results of further exploratory analyses (all 2-tailed). There was a significant positive correlation between overall action binding on ketamine and overall CADSS score (*ρ*=.61, *p*=.035) and also ‘Unreality’ (*ρ*=.59, *p*=.044). There were no other significant correlations. There was, however, a near-significant correlation between overall action binding on ketamine and ‘Memory’ (*ρ*=.57, *p*=.05) and also a near-significant negative correlation between predictive action binding on ketamine and ‘Memory’ (*ρ*=−.55, *p*=.06).

### 3.3 Control analyses

We also performed several control analyses. In a first analysis we compared the mean standard deviation of time estimates across all trials on ketamine vs. placebo. High standard deviations reflect high variability in time estimates, indicating possible difficulty with the intentional binding task (Moore et al., 2010). We predicted that ketamine would increase the variability in time estimates, so we used a 1-tailed test of significance. This analysis showed a significant difference in the mean standard deviation on ketamine (mean: 86.09 ms) vs. placebo (mean: 66.91 ms), *t*(11)=1.96, *p*=.04. Although this suggests that ketamine caused more difficulty with the task we do not think that this can explain the *pattern* of results with respect to differences in prediction on ketamine vs. placebo. In particular, it is not clear how an increase in variability would lead to an increased sensitivity to the probability manipulation and a concomitant increase in binding on ‘action only’ trials in the high outcome probability condition. If there was a general effect of the drug on timing, we would have expected a significant increase in binding on both trial types.

In a second control analysis we considered the effect of drug session order (i.e., placebo session first vs. ketamine session first). It may be that the significant increase in predictive binding on ketamine was driven by a certain order of testing. We compared predictive binding on ketamine vs. placebo (‘Drug’ factor), introducing ‘Drug session order’ as a *between subjects* factor. As expected, there was a significant main effect of ‘Drug’, *F*(1, 10)=5.18, *p*=.046. Crucially, there was no significant interaction between ‘Drug’ and ‘Drug session order’, *F*(1, 10)=.18, *p*=.68. This suggests that drug order was not responsible for the effects observed in this study.

In a final control analysis we considered the effect of block order (i.e., 50% condition first vs. 75% condition first). It may be that the significant increase in predictive binding on ketamine was driven by a certain order of testing. For each drug session we compared the magnitude of predictive action binding as a function of block order. There was no significant difference in the magnitude of this effect as function of block order on ketamine (*t*(10)=.96, *p*=.36) or placebo (*t*(10)=.48, *p*=.64) (2-tailed). This suggests that block order was not responsible for the effects observed in this study.

## 4 Discussion

Sense of agency is associated with systematic changes in the subjective experience of time (Haggard et al., 2002; Moore & Haggard, 2010; Moore & Obhi, 2012), an effect known as ‘intentional binding’. We investigated the. impact of ketamine, an important drug model for schizophrenia, on the action component of this effect. In replication of a previous result (Moore et al., 2011), it was found that ketamine significantly increased the magnitude of overall action binding. Moreover, the drug significantly increased the predictive contribution to action binding, an effect which closely resembles the performance of patients with prodromal symptoms of schizophrenia, reported in a previous study (Hauser et al., 2011). Critically, too, we demonstrated significant relationships between the effects of ketamine on this behavioural binding effect and the psychotomimetic effects of the drug.

### 4.1 Hyper-binding and hyper-prediction: A common role of prediction error?

The increase in overall action binding on ketamine relative to placebo replicates a previous finding (Moore et al., 2011). Importantly, the magnitude of binding in patients with schizophrenia is similarly increased relative to controls (Haggard et al., 2003; Voss et al., 2010). This increase in binding therefore appears to be a robust cognitive aspect of psychotic illness, and one that ketamine is able to reproduce reliably.

Of central interest in the present study was the effect of ketamine on predictive action binding. In healthy adults, a strong expectation that an action will produce an outcome is sufficient to generate action binding (Moore & Haggard, 2008; Moore, Lagnado, Deal, & Haggard, 2009). Compared to controls, predictive action binding is reduced in patients with schizophrenia (Voss et al., 2010), whereas it is increased in prodromal patients (Hauser et al., 2011). In the present study we found that ketamine significantly increased the magnitude of predictive action binding, an effect that is most redolent of the prodromal stage of the illness.

We have previously suggested that the overall increase in binding may be linked to inappropriate prediction error signalling (Moore et al., 2011). Prediction error refers to the mismatch between expectation and occurrence, and is used as a teaching signal to drive causal associations between events (Dickinson, 2001). Aberrant or inappropriately persistent error signalling is observed in patients with schizophrenia (Corlett, Honey, and Fletcher 2007; Murray et al., 2008; Schlagenhauf et al., 2009; Fletcher & Frith, 2009) and also following ketamine administration in healthy volunteers (Corlett et al., 2007, 2006; Corlett, Honey, Krystal, & Fletcher, 2010). Since error is a signal to strengthen causal associations, persistent signalling of error in schizophrenia and following ketamine administration would be expected to inappropriately strength action-outcome association, resulting in the observed hyper-binding. One further possibility is that the hyper-prediction found in the present study (and in prodromal patients) is a consequence of hyper-binding, with strong action-outcome associations forming the basis of future outcome predictions. We are cautious however, in this speculation, given that the known impact of ketamine on prediction error signalling could also produce the opposite effect, rendering the experimentally manipulated contingencies less clear in the drug-treated state. It is also worth noting that certain studies encourage the opposite prediction. That is, prediction error could *reduce* the strength of action-outcome associations (e.g., Elsner & Hommel, 2004), which would reduce the strength of prediction. Moreover, some studies on intentional binding itself have shown that (temporal) unpredictability reduces the binding effect (Haggard et al., 2002). Future investigations should directly consider the effect of prediction error on intentional binding, given the current uncertainty.

### 4.2 Correlations between binding and CADSS

Our correlation analyses suggest that the effects of ketamine on intentional binding are closely related to the psychotomimetic effects of the drug. Although not quite significant, the positive correlation between overall action binding on ketamine and scores on the ‘Body Perception’ subscale is consistent with a previous result (Moore et al., 2011). Taken together these results imply a close connection between the sense of agency and the experience of one’s own body (the ‘sense of ownership’). That agency and ownership are so entwined has been recognised in previous philosophical (Gallagher, 2000; Tsakiris, Schütz-Bosbach, & Gallagher, 2007) and psychological (Tsakiris, Prabhu, & Haggard, 2006) investigations. The results of the current study also suggest that disturbances in this agency-ownership relationship may be a core feature of psychotic illness.

### 4.3 Sense of time and sense of agency: Common neurochemical bases?

The well-established relationship between intentional binding and sense of agency suggests an intimate link between our experience of the temporal characteristics of our actions and our sense that we are the authors of these actions. While the precise nature of this relationship is unclear, it is noteworthy that schizophrenia, which, as we have described, is associated with profound alterations in sense of agency, also entails an impairment in temporal estimation (e.g., Rammsayer, 1990). This supports the proposed relationship between subjective timing and agency, an observation consistent with the fact that key neurotransmitters thought to be disrupted in schizophrenia (dopamine and glutamate) are also implicated in timing (Meck, 1996) and SoA (Moore et al., 2010, 2011). Furthermore, we have recently shown that acute administration of ketamine, which can distort the experience of time in healthy volunteers (Pomarol-Clotet et al., 2006), produces selective deficits in a task evaluating the ability to compare successive temporal durations (Coull et al., 2011). This suggestion is further supported by the fact that regions thought to underpin sense of agency, such as the supplementary motor cortices and basal ganglia, also underpin time perception.

Given this evidence for a link between sense of agency and the subjective timing of internally-generated and externally experienced events, we should consider the possibility that the effects of ketamine in the current study simply be related to a generic perturbation of the ability to make temporal estimations, either through direct effects on timing mechanisms or indirectly through effects on attention. We do not believe that this is so, given that action time was normalised to a baseline time estimate. Were the deficit to be non-specific it is unlikely that it would affect one condition and not the other. Furthermore, the effect of ketamine was selective, producing a significant increase in binding on ‘action only’ trials in the 75% vs. 50% conditions. Finally, previous studies using even higher doses of ketamine suggest that participants are able to perform attention and working memory tasks without difficulty (Honey et al., 2004, 2003). In this way, a general effect on attention and/or timing is unlikely to explain our results.

Finally, an outstanding issue concerning intentional binding is whether the sense of agency is a cause or a consequence of the subjective compression of time between actions and their effects. More experimental work is needed to clarify this relationship. However, an intriguing hypothesis (Stetson, Cui, Montague, & Eagleman, 2006) is that sense of agency is the cause (rather than the consequence). According to this view, we expect that outcomes caused by our own actions are temporally contiguous. Once we recognise that an outcome is contingent on our own behaviour (i.e., we have a *sense of agency* for it), then a recalibration mechanism is engaged, bringing these two events closer together in subjective time. In this way, the sense of agency triggers a temporal contiguity prior that pulls actions and outcomes together in subjective time. This suggests that perception of time, as with other perceptions, may be strongly modulated by prior expectancy. In this respect, the current findings might run counter to our previous suggestion (Corlett, Frith, & Fletcher, 2009; Corlett et al., 2010) that ketamine’s effects in part arise from a weakening of feedback modulation and hence an attenuated impact of prior expectations on current input.

### 4.4 Limitations of the study

Certain limitations of the study must be acknowledged. On placebo we failed to find a significant predictive contribution to action binding. This limitation is perhaps explained by the within-subjects design: given that each participant experienced action-outcome pairings at varying contingencies, then it is possible that predictions were less strong than they would otherwise have been. Furthermore, our experimental procedure was necessarily shorter than previous binding studies owing to time constraints inherent in drug studies. As described above, one effect of ketamine may be to artificially augment the magnitude of PE signals. This would mean that the rate of learning is faster on ketamine compared with placebo. Given this, one would expect the magnitude of binding, and the influence of prediction, on placebo to be attenuated in this shortened version of the task. This is precisely what we observed. Finally, the magnitude of the predictive contribution to action binding on ketamine (24 ms) was of a very similar magnitude to prodromal patients (27 ms; Hauser et al., 2011). This shows that ketamine produces a strikingly similar predictive abnormality.

The limitations of the ketamine drug model of schizophrenia should also be acknowledged. For example, whilst ketamine produces a range of symptoms associated with endogenous psychosis (arguably a broader range than other drug models of the disease; Krystal et al., 1994) there are notable exceptions (Fletcher & Honey, 2006). Furthermore, ketamine produces changes that are not necessarily associated with schizophrenia, such as euphoria (Fletcher & Honey, 2006). Indeed, we would argue that ketamine actually presents a very limited model of established schizophrenia, rather more compellingly reproducing the early/prodromal symptoms, a suggestion in keeping with the current findings. Despite these limitations of the ketamine drug model, we do not think they significantly undermine our interpretation of the present data. We have shown, once again, that ketamine boosts overall action binding, replicating the findings of a previous study (Moore et al., 2011). This effect is also consistently observed in patients (Haggard et al., 2003; Voss et al., 2010). Furthermore, the pattern of predictive action binding in healthy volunteers on ketamine is entirely consistent with that found prodromal patients.

In this paper we have emphasised the importance of sensorimotor prediction for binding and the sense of agency. However, it should be noted that information from *various* sources is likely to be involved (Moore, Wegner, & Haggard, 2009; Synofzik, Vosgerau, & Newen, 2008; Wegner & Sparrow, 2004). For example, Daniel Wegner and colleagues have shown that the experience of agency can be established even in the absence of movement (Moore et al., 2009; Wegner, Sparrow, & Winerman, 2004). In light of this, it has been suggested that processes involving sensorimotor prediction are unable to *fully* explain the sense of agency. Instead, they may be limited to lower level, implicit aspects of this experience, which is what intentional binding may be closer to.

Related to this, there is an ongoing debate concerning the neurocognitive origins of intentional binding. For example, some have emphasised the importance of sensorimotor prediction (Haggard et al., 2002; Moore & Haggard, 2008), whereas others have suggested that intentional binding depends on more general predictive processes (e.g., Desantis, Roussel, & Waszak, 2011.). We would suggest that both low level sensorimotor prediction and higher level conscious expectation are likely to be important and that their relative influence will be shaped by factors such as context and cue reliability. This would be consistent with recent optimal cue integration approaches which recognise the importance of various sources of information for intentional binding and sense of agency (Moore & Fletcher, 2012).

Finally, it has been suggested that intentional binding is related to causality more generally rather than agency specifically. However, there is little evidence to directly support this. Some studies have shown that causality is a necessary condition for intentional binding (e.g., (Buehner & Humphreys, 2009; Moore et al., 2009). However, no studies have, to our knowledge, shown that it is sufficient (see Moore & Obhi, 2012, for discussion). Another possible source of evidence comes from studies showing that binding can occur in the absence of voluntary action (e.g., Dogge et al., 2012; Moore et al., 2009). However, these effects depend upon *implied self*-causation or the modification intentional content prior to the movement, both of which are highly relevant to agency, rather than causality more generally.

### 4.5 Conclusion

Despite the aforementioned caveats, the present study provides strong evidence that ketamine may best reproduce a state resembling the psychotic prodrome, rather than established schizophrenic illness. Using a measure of agency based on the subject experience of time we found that ketamine engendered excessively strong sensorimotor predictions. This closely resembles previous data from prodromal patients. Given the concordance between the effects of ketamine and prodromal patients, we suggest that this further supports the use of ketamine as a tool to explore the genesis of psychotic illness.

## Figures and Tables

**Table 1:** Mean binding effects (ms) for each drug session (SD across subjects in parentheses). Postdictive action binding is calculated by subtracting binding on ‘action only’ trials from binding on ‘action+tone’ trials in the 50% condition. The more positive this difference the stronger the postdictive effect. Predictive binding, the focus of this study, is calculated by subtracting binding on ‘action only’ trials in the 50% condition from binding on the same trials in the 75% condition. The more positive this difference the stronger the predictive effect.

Drug session	Condition (%)	Trial type	Action binding (ms) (SD)	Postdictive action binding (ms) (SD)	Predictive action binding (ms) (SD)
Placebo	50	Action only	−2.0 (30)		
		Action+tone	6.0 (30)	8.0 (27)	
	75	Action only	−14.0 (42)		−12.0 (48)
		Action+tone	2.0 (30)		
Ketamine	50	Action only	1.0 (31)		
		Action+tone	−4.0 (33)	−5.0 (31)	
	75	Action only	25.0 (21)		24.0 (38)
		Action+tone	34.5 (22)		
